# Salubrinal Alleviates Collagen-Induced Arthritis through Promoting P65 Degradation in Osteoclastogenesis

**DOI:** 10.3390/ijms22073501

**Published:** 2021-03-28

**Authors:** Ziyu Wang, Zijian Li, Guojue Wang, Ying Sun, Yuanyang Yuan, Hong Nie

**Affiliations:** Department of Immunology and Microbiology, Shanghai Institute of Immunology, Shanghai Jiao Tong University School of Medicine, Shanghai 200025, China; ziyuwang@sjtu.edu.cn (Z.W.); lizijian0706@163.com (Z.L.); gjwang0422@sjtu.edu.cn (G.W.); sunyingying@sjtu.edu.cn (Y.S.)

**Keywords:** Salubrinal, collagen-induced arthritis, osteoclast, NF-κB signaling pathway, P65

## Abstract

Rheumatoid arthritis (RA) is a complex systemic autoimmune disorder that primarily involves joints, further affects the life quality of patients, and has increased mortality. The pathogenesis of RA involves multiple pathways, resulting in some patients showing resistance to the existing drugs. Salubrinal is a small molecule compound that has recently been shown to exert multiple beneficial effects on bone tissue. However, the effect of Salubrinal in RA has not been clearly confirmed. Hence, we induced collagen-induced arthritis (CIA) in DBA/1J mice and found that Salubrinal treatment decreased the clinical score of CIA mice, inhibiting joint damage and bone destruction. Furthermore, Salubrinal treatment downregulated osteoclast number in knee joint of CIA in mice, and suppressed bone marrow-derived osteoclast formation and function, downregulated osteoclast-related gene expression. Moreover, Salubrinal treatment inhibited RANKL-induced NF-κB signaling pathway, and promoted P65 degradation through the ubiquitin-proteasome system, further restrained RANKL-induced osteoclastogenesis. This study explains the mechanism by which Salubrinal ameliorates arthritis of CIA in mice, indicating that Salubrinal may be a potential drug for RA, and expands the potential uses of Salubrinal in the treatment of bone destruction-related diseases.

## 1. Introduction

Rheumatoid arthritis (RA) is a chronic progressive autoimmune disorder that typically causes cartilage structural damage, synovial inflammation, and bone erosion, resulting in swelling and destruction of the joints and corresponding disability [1]. Bone loss in RA results from excessive osteoclast activation and blunted bone formation, which is correlated with disease severity and functional disturbance [2]. Conventional synthetic disease-modifying antirheumatic drugs (DMARDs) alleviate disease progression in some RA patients, although a comprehensive perspective of the mechanisms of action of these drugs remains elusive [3]. Biologic DMARDs (bDMARDs) represented by TNF inhibitors have revolutionized the treatment landscape for RA in the past few decades [4]. However, several clinical studies have shown that refractory disease still presents a significant clinical challenge to multiple bDMARDs [5]. In the past few years, the oral small-molecule inhibitors, such as Janus kinase inhibitors (such as Tofacitinib) and nuclear factor (NF)-κB inhibitors (such as Iguratimod), have improved treatment outcomes in patients with RA by interfering with various intracellular signaling pathways upon which immune functions converge [6,7]. Thus, these drugs have been shown to have efficacies similar to those of bDMARDs in the treatment of RA.

Bone homeostasis is regulated by osteoclast-mediated bone resorption and osteoblast-mediated bone formation [8]. Osteoclasts are the only cells responsible for bone resorption in vivo that are derived from hematopoietic precursor cells. Macrophage-colony stimulating factor (M-CSF) and receptor activator of nuclear factor-κB ligand (RANKL) are required for osteoclast proliferation, differentiation, and activation. M-CSF supports osteoclast precursors’ survival and proliferation by binding to M-CSF receptor c-Fms on osteoclast precursors. Meanwhile, RANKL binds to its receptor RANK to trigger multiple signaling transduction pathways in osteoclast precursors, promoting the differentiation of osteoclast precursors into osteoclasts. Then, mature osteoclasts experiencing cytoskeletal rearrangements and spreading in a c-Src dependent manner in response to M-CSF and RANKL [9,10,11], finally adhere to bone matrix as multinucleated osteoclasts. Acid and lytic enzymes are secreted by RANKL-activated multinucleated osteoclasts to degrade bone matrix in a specialized compartment, and then can cause cartilage and bone damage in vivo. In RA, osteoclasts show enhanced differentiation capacity and increased activity related to direct intercellular interactions, and the systemic effects of inflammatory cytokines and autoantibodies involved in RA [12]. Furthermore, osteoclasts are much more than bone decomposers. Increasing evidence indicates that several pathogens use osteoclasts as a host cell to reproduce and escape, finally causing bone damage [13]. Thus, osteoclasts are also a new cell target for bone defects caused by infections.

Salubrinal is a small compound with a molecular weight of 479.81 that rescues rat PC12 cells from endoplasmic reticulum stress-induced apoptosis by inhibiting the dephosphorylation of phospho-eIF2α via control of serine/threonine protein phosphatase 1 and growth arrest- and DNA damage-induced transcript 34 [14]. Through regulation of the eIF2α-mediated signaling pathway, Salubrinal exerts various beneficial effects on bone metabolism, including the inhibition of matrix metalloproteinase (MMP) 13 expression and activity in chondrocytes via the inactivation of p38 and NF-κB signaling pathways [15] and activation of activating transcription factor 4 translation by elevation of eIF2α phosphorylation, and hence promotes osteoblastogenesis and bone regeneration [16]. Moreover, Salubrinal suppresses bone resorption by inhibiting RANKL-induced osteoclastogenesis through inactivation of nuclear factor of activated T-cells, cytoplasmic, calcineurin dependent 1 (NFATc1) [17]. Briefly, Salubrinal has potential to be a candidate drug in skeletal system disease by promoting osteoblastogenesis, inhibiting osteoclastogenesis, and suppressing chondrocytes activity. It was reported that Salubrinal could suppress inflammation in anti-collagen antibody-induced arthritis (CAIA) by inhibiting dual-specificity phosphatase 2 [18]. However, the effect and mechanism of action of Salubrinal in RA have not been clearly confirmed.

Therefore, in this study, we first investigated the potential therapeutic effects of Salubrinal in a mouse model of collagen-induced arthritis (CIA), a classical arthritis model that resembles human RA. We further explored the mechanism by which Salubrinal inhibits osteoclast formation. We hope to thus find a new treatment strategy for RA and other diseases with osteoclast-related bone destruction.

## 2. Results

### 2.1. Salubrinal Reduced Disease Severity in CIA Mice

**Figure 1:**
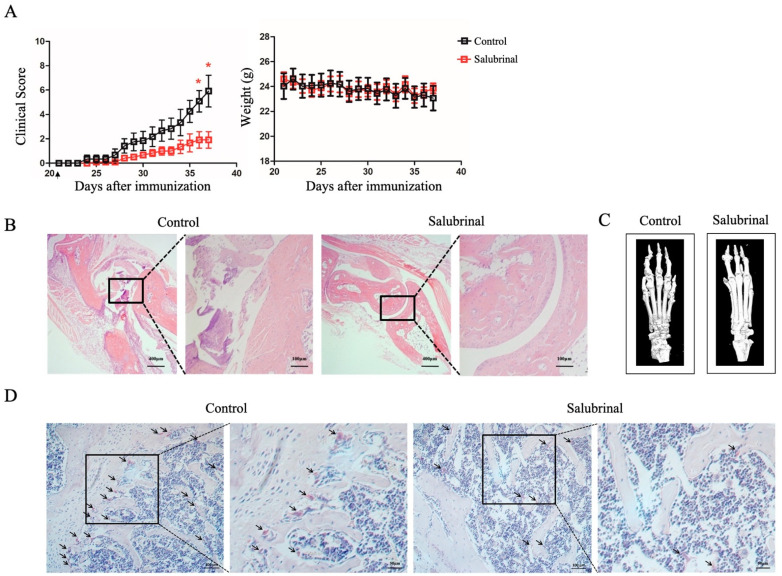
Salubrinal alleviated the clinical symptoms of collagen-induced arthritis (CIA) mice. CIA was induced in DBA/1J mice by subcutaneous injection with CII on day 0 and day 21. Mice were injected intraperitoneally with vehicle or Salubrinal daily beginning on day 21 and were sacrificed on day 37. (**A**) Clinical score for arthritis and body weight (*n* = 6/group). (**B**) Representative H&E staining of ankle joint sections. (**C**) Representative three-dimensional renditions of the ankle joints and paws using micro-CT. (**D**) Tartrate-resistant acid phosphatase (TRAP) staining on the knee joints. Arrows indicate wine red areas. Data are shown as means ± SEM. * *p* < 0.05.

First, we established a CIA model in DBA/1J mice. Daily Salubrinal (2 mg/kg) treatment was started on day 21. The results showed that Salubrinal markedly attenuated the severity of arthritis, but did not affect the body weights of mice (Figure 1A). Additionally, decreased synovium inflammation and joint damage were observed in the CIA mice treated with Salubrinal (Figure 1B). Furthermore, we observed that Salubrinal reduced the degree of bone destruction through micro-CT and three-dimensional reconstruction of the ankle joints and paws (Figure 1C). As osteoclasts are the only cells responsible for bone resorption in vivo, we next evaluated the effects of Salubrinal on osteoclast formation in the knee joints of CIA mice by tartrate-resistant acid phosphatase (TRAP) staining. Our results showed that the number of osteoclasts decreased after treatment with Salubrinal (Figure 1D). Overall, these data showed that Salubrinal inhibited joint damage, thereby alleviating the clinical symptoms of CIA in mice.

### 2.2. Salubrinal Inhibited Osteoclast Formation In Vitro

**Figure 2:**
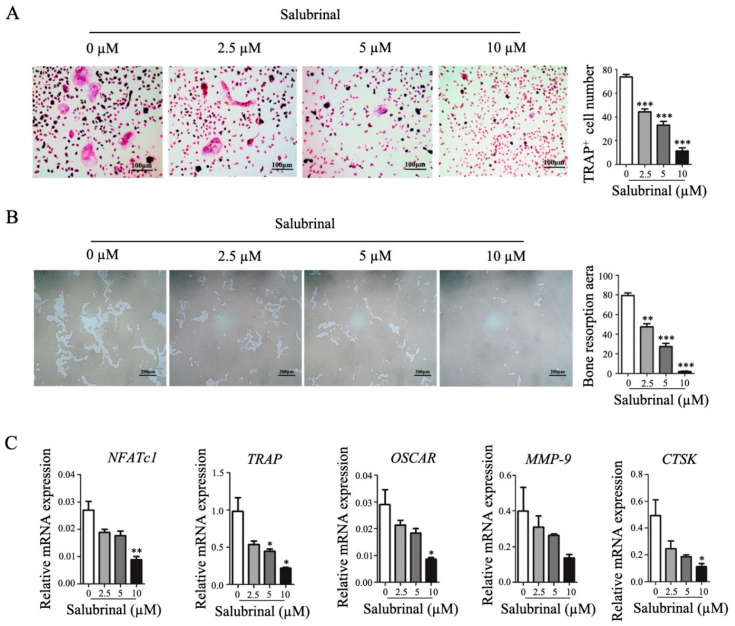
Salubrinal suppressed osteoclast formation and function. To induce osteoclast differentiation, bone marrow-derived cells were treated with M-CSF for 3 days, and with RANKL and M-CSF for another 4 days. Salubrinal was added together with RANKL for 4 days. (**A**) Osteoclast numbers were quantified by TRAP staining. (**B**) Osteoclast bone-resorption ability was investigated using hydroxyapatite-coated OsteoAssay plate resorption assays. (**C**) *NFATc1*, *TRAP*, *OSCAR*, *MMP-9*, and *CTSK* mRNA expression levels were detected by qPCR. Data are shown as means ± SEM. * *p* < 0.05, ** *p* < 0.01, *** *p* < 0.001 (Salubrinal treatment groups vs. none treated group).

To further investigate the effects of Salubrinal on osteoclastogenesis in vitro, bone marrow cells were separated and differentiated into osteoclasts by stimulation with M-CSF and RANKL. We found that Salubrinal decreased osteoclast number in a dose-dependent manner, as indicated by TRAP staining (Figure 2A). Next, we investigated the effects of Salubrinal on osteoclast function using bone resorption assays. The results showed that Salubrinal inhibited hydroxyapatite coating-removal (as a surrogate for bone resorption) mediated by osteoclasts in a dose-dependent manner (Figure 2B). Consistent with these results, genes related to osteoclast formation and function (such as *NFATc1, CTSK*, etc.) were also downregulated by Salubrinal in a dose-dependent manner (Figure 2C). Thus, our findings showed that Salubrinal could suppress osteoclast formation and function.

### 2.3. Salubrinal Suppressed RANKL-Induced NF-κB Signaling Pathway

**Figure 3:**
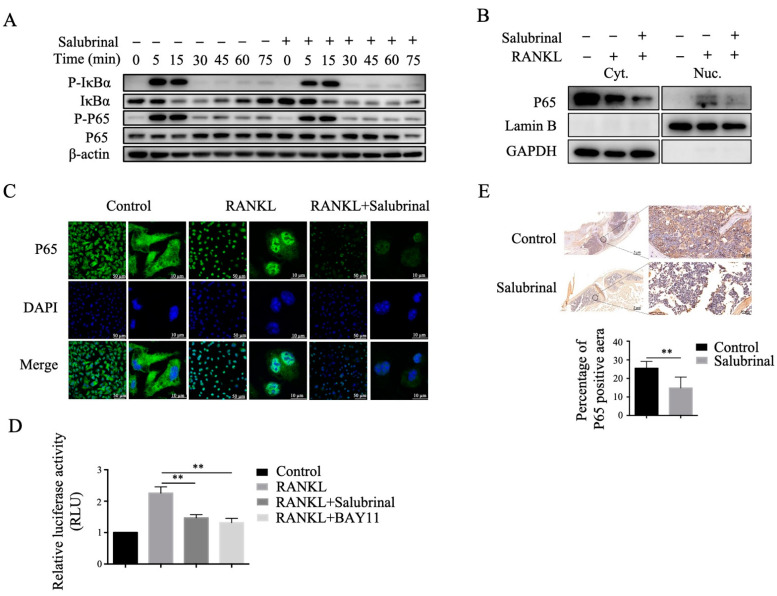
Salubrinal downregulated P65 abundance and inhibited the RANKL-induced NF-κB signaling pathway. (**A**) Phospho-IκBα, IκBα, phospho-P65, and P65 expression levels were analyzed by Western blotting after stimulation with RANKL (30 ng/mL) for the indicated times in bone marrow-derived osteoclast precursors pretreated with Salubrinal (10 µM) for 3 h. P65 abundance in the nucleus and cytoplasm was analyzed by Western blotting (**B**) and immunofluorescence staining (**C**) after stimulation with RANKL (30 ng/mL) for 30 min in bone marrow-derived osteoclast precursors pretreated with Salubrinal (10 µM) for 3 h. (**D**) NF-κB signaling transcriptional activity was measured using dual-luciferase reporter assays. (**E**) P65 abundance in knee joints of CIA mice was detected by immunohistochemical staining. Data are shown as means ± SEM. ** *p* < 0.01.

The RANKL-induced NF-κB signaling pathway is a vital pathway involved in osteoclastogenesis and osteoclast function [19]. Therefore, we investigated the effects of Salubrinal on the RANKL-induced NF-κB signaling pathway in osteoclast precursors by Western blotting. We found that Salubrinal treatment decreased the resynthesis abundance of IκBα and downregulated the protein level of P65, a key transcription factor of IκBα in the NF-κB pathway (Figure 3A). Moreover, we found that after RANKL stimulation, P65 abundance was decreased in the cytoplasm and nucleus of osteoclast precursors by Salubrinal treatment (Figure 3B). This result was further confirmed using immunofluorescence technique (Figure 3C). In addition, we found that Salubrinal inhibited RANKL-induced NF-κB signaling pathway transcriptional activity, similar to the effects of the NF-κB inhibitor BAY-11 (Figure 3D). According to the above results, we deduced that Salubrinal inhibited the RANKL-induced NF-κB signaling pathway by decreasing P65 protein level. Further we found Salubrinal treatment in vivo also decreased P65 expression in the knees of CIA mice (Figure 3E). Overall, these data suggested that Salubrinal might inhibit the RANKL-induced NF-κB signaling pathway by downregulating P65 abundance.

### 2.4. Salubrinal Inhibited Osteoclast Formation by Downregulating P65 Abundance

**Figure 4:**
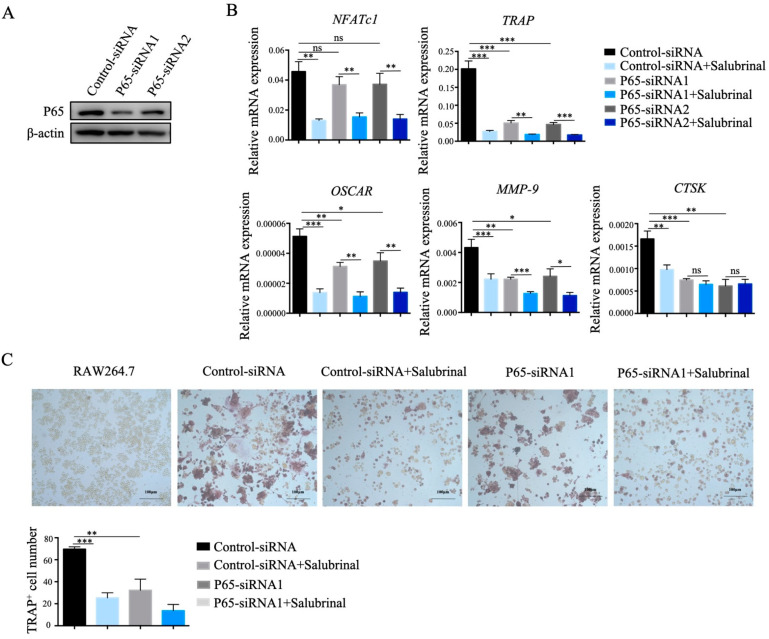
Salubrinal inhibited osteoclast formation by downregulating P65. We transfected specific P65-siRNA in RAW264.7 cells by using Attractene Transfection Reagent to knockdown of P65. (**A**) P65 abundance in RAW264.7 cells after P65 knockdown. (**B**) mRNA expression levels of *NFATc1*, *OSCAR*, *TRAP*, *MMP-9*, and *CTSK* gene were detected by qPCR after induction by RANKL (100 ng/mL) for 24 h, Salubrinal (10 µM) was added together with RANKL for 24 h. (**C**) Osteoclast numbers were quantified by TRAP staining after induction by RANKL (100 ng/mL) for 4 days, Salubrinal (10 µM) was added for the last 3 days. Data are shown as means ± SEM. * *p* < 0.05, ** *p* < 0.01, *** *p* < 0.001.

To determine whether Salubrinal impaired osteoclastogenesis by downregulating P65 protein level, we designed two pairs of siRNA oligonucleotides specific for *P65* mRNA and used them to transfect RAW264.7 cells, resulting in P65 knockdown (Figure 4A). After P65 knockdown, the mRNA expression levels of *TRAP*, *matrix metalloproteinase 9 (MMP-9),* and *cathepsin K (CTSK)* gene were significantly reduced and comparable with Salubrinal treatment, whereas *osteoclast-associated receptor (OSCAR)* gene expression levels were partly decreased, and *NFATc1* expression levels were not significantly influenced. However, the expression levels of both genes were obviously decreased after Salubrinal treatment (Figure 4B). These results indicated that knockdown of P65 had an effect similar to that of Salubrinal treatment in the regulation of osteoclast-regulated gene expression. Finally, we induced P65-knockdown RAW264.7 cells to differentiate into osteoclasts and found that osteoclast formation was significantly suppressed, comparable to the levels achieved with Salubrinal treatment (Figure 4C). Taken together, these findings indicated that Salubrinal inhibited osteoclast formation by downregulating P65 protein level.

### 2.5. Salubrinal Downregulated P65 Expression by Promoting P65 Degradation

**Figure 5:**
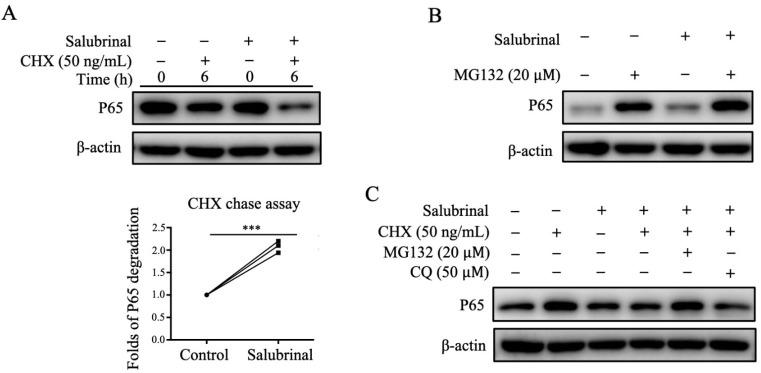
Salubrinal promoted P65 degradation through the ubiquitin-proteasome system. Bone marrow-derived osteoclast precursors were pre-incubated with Salubrinal (10 µM) for 3 h, induced with RANKL (30 ng/mL) for 30 min, washed with PBS, and treated with CHX (**A**), MG132 (**B**), or CHX + MG132/CQ (**C**) for 6 h. The abundance of p65 protein was analyzed by Western blotting. Data are shown as means ± SEM. *** *p* < 0.001.

To study the mechanism involved in the decrease in P65 protein level induced by Salubrinal treatment, we used *CHX* chase experiments to check how Salubrinal affected P65 total protein abundance. After using CHX to inhibit protein synthesis, P65 abundance was still downregulated by Salubrinal (Figure 5A), indicating that Salubrinal decreased P65 abundance by promoting P65 degradation. Additionally, treatment with the proteasome inhibitor MG132 and the autophagy inhibitor CQ showed that Salubrinal promoted P65 degradation through the ubiquitin-proteasome system (Figure 5B) rather than the autophagy-lysosome pathway (Figure 5C). Taken together, our results indicated that Salubrinal mediated P65 degradation is dependent on the ubiquitin-proteasome system.

## 3. Discussion

In this study, we demonstrated that Salubrinal could alleviate the clinical symptoms of CIA in mice by reducing bone erosion and joint destruction. Moreover, Salubrinal inhibited the RANKL-induced NF-κB signaling pathway by promoting P65 degradation via the ubiquitin-proteasome system, further suppressing RANKL-induced osteoclastogenesis.

Previous studies on Salubrinal have focused on its neuroprotective effects against neurotoxic substances, demonstrating that this drug can mediate neurological disorders via inhibition of eIF2α dephosphorylation [20]. However, in recent studies, Salubrinal was shown to exert multiple beneficial effects on skeletal tissues, including effects on chondrocytes, osteoclasts, and osteoblasts. Although it was reported that Salubrinal could suppress inflammation in anti-collagen antibody-induced arthritis (CAIA) by inhibiting dual-specificity phosphatase 2 [18], the CAIA model does not fully simulate the pathogenesis of RA because CAIA mice exhibit arthritis triggered by passive immunity rather than active immunity.

In this study, we proved that Salubrinal could alleviate the clinical symptoms of CIA in mice by reducing bone erosion and joint destruction. Furthermore, we observed that Salubrinal decreased osteoclast number in the joint of CIA mice. Osteoclasts are specialized cells derived from the monocyte/macrophage hematopoietic lineage that adhere to the bone matrix and then secrete acid and lytic enzymes that can cause cartilage and bone damage in RA [21]. Two cytokines, RANKL and M-CSF, are both necessary for osteoclastogenesis. These proteins act together to induce the expression of genes related to osteoclast differentiation and function, leading to the development of mature osteoclasts [22]. In our study, we used RANKL to induce osteoclastogenesis and found that Salubrinal decreased RANKL-induced bone marrow-derived osteoclast number and function and reduced the expression levels of various genes, including *NFATc1*, *TRAP*, *OSCAR*, and *CTSK*, which are the markers of the osteoclast lineage and are responsible for osteoclast function [23,24,25]. Among these genes, *NFATc1* is the first to receive RANKL signals. RANKL-induced osteoclast differentiation is divided into three stages. First, RANKL binds to RANK, resulting in the recruitment of TRAF6 and leading to the activation of the NF-κB signaling pathway. NF-κB and NFATc2 are then recruited to the *NFATc1* promoter at an early stage. Second, activated NFATc1 binds to its own promoter with activating protein-1 (AP-1), leading to the autoamplification of *NFATc1*. In this stage, AP-1 is critical for *NFATc1* autoamplification. Third, a number of osteoclast-specific genes, including *CTSK*, *TRAP*, and MMP-9, are activated by NFATc1 and other transcriptional factors. Therefore, the NF-κB and AP-1 signaling pathways are vital in RANKL-induced osteoclast differentiation, and inhibition of IKKβ and IKKα in RANKL-induced bone marrow-derived osteoclast precursors could suppress osteoclastogenesis and prevent inflammatory bone destruction [26].

Previous studies have indicated that Salubrinal can inhibit RANKL-induced AP-1 signaling in RAW264.7 cells [17,27]. Here, we showed that Salubrinal inhibited the RANKL-induced NF-κB signaling pathway by downregulating P65 protein abundance in bone marrow-derived osteoclast precursors. Furthermore, after P65 knockdown, RANKL-induced osteoclastogenesis was significantly suppressed. P65 knockdown also decreased *TRAP*, *MMP-9*, and *CTSK* expression levels, which were comparable to those under Salubrinal treatment. However, *NFATc1* expression levels were not significantly affected by P65 knockdown, and *OSCAR* expression levels were partly decreased by P65 knockdown, whereas Salubrinal significantly inhibited the expression of the both genes. We speculate that this may be because P65 is recruited to the *NFATc1* promoter at an early stage, as described above. AP-1 facilitates the autoamplification of *NFATc1*, and the transcription of *OSCAR* is mainly controlled by NFATc1 [25]. Thus, P65 knockdown can have early inhibitory effects on *NFATc1* transcription, whereas NFATc1 can still undergo autoamplification without inhibition of AP-1. However, Salubrinal inhibited both the NF-κB and AP-1 pathways. Therefore, the effects of Salubrinal on suppression of RANKL-induced osteoclastogenesis relied on the inhibition of both the NF-κB and AP-1 pathways.

Notably, Salubrinal promoted P65 degradation through the ubiquitin-proteasome system rather than the autophagy-lysosome pathway. Additionally, we found that Salubrinal could directly bind to P65, which may influence the binding of P65 to the proteasome and is responsible for promoting P65 degradation (data not shown). NF-κB is a critical transcription factor that regulates multiple immune and inflammatory responses, and the most abundant form of NF-κB in the classical pathway is the heterodimer of p50 and P65 [28]. An increase in activated P65 levels triggers the overactivation of downstream effector pathways that are involved in many autoimmune diseases [29]. Hence, the NF-κB P65 signaling pathway is essential for drug discovery. Moreover, P65 degradation has been suggested as a mechanism that controls the strength and duration of NF-κB activation [30]. Thus, our findings highlight the potential for targeting P65 using Salubrinal, suggesting promising applications of Salubrinal in the clinical setting.

Taken together, our findings demonstrated that Salubrinal could serve as an efficient therapeutic drug for CIA by inhibiting osteoclast formation and function. Our results also clarified the potential mechanisms, showing that Salubrinal suppressed RANKL-induced NF-κB signaling by downregulating P65 protein abundance via promotion of P65 degradation by the ubiquitin-proteasome system. Our findings established a solid foundation for the application of Salubrinal as a potential treatment for RA and expanded the potential uses of Salubrinal in the treatment of bone destruction-related diseases.

## 4. Materials and Methods 

### 4.1. Mice and Reagents 

Eight-week-old male DBA/1J mice and six-week-old male C57BL/6 mice (Shanghai SLAC Laboratory Animal Co., Ltd., Shanghai, China) were maintained under pathogen-free conditions at Shanghai Jiao Tong University School of Medicine. All experimental procedures were performed in accordance with the guidelines of the Animal Care and Use Committee. Salubrinal and MG132 were purchased from Selleck Chemicals (Houston, TX, USA). BAY11 was purchased from Beyotime Biotechnology (Shanghai, China), and cycloheximide (CHX) was purchased from MedChemExpress (Monmouth Junction, NJ, USA). Chloroquine (CQ) was purchased from Sigma-Aldrich (St. Louis, MO, USA).

### 4.2. Induction and Treatment of CIA 

To establish the CIA model, eight-week-old male DBA/1J mice were intradermally injected with CII (150 μg/mouse; Chondrex, Redmond, WA, USA) mixed with Freund’s complete adjuvant (Sigma-Aldrich, St. Louis, MO, USA) in the tail on day 0. On day 21, mice were injected with CII (75 μg/mouse) mixed with Freund’s incomplete adjuvant (Sigma-Aldrich, St. Louis, MO, USA) as a booster. On the day of the booster injection, the mice were randomly divided into two groups, and intraperitoneally injected with Salubrinal (2 mg/kg, stocked in dimethyl sulfoxide, further dissolved in phosphate-buffered saline (PBS)) or with dimethyl sulfoxide (dissolved in PBS), respectively. Mice were observed daily and scored for disease severity as previous describe [31].

### 4.3. Histochemical Analysis and Micro-Computed Tomography (Micro-CT)

CIA mice were sacrificed on day 37 after the first immunization. The hind legs were fixed, and three-dimensional micro-CT analysis was performed. Micro-CT scanning was performed using a SkyScan1176 instrument as previous described [32]. Three-dimensional microstructural image data were analyzed using CT VOX software (Skycan). Alternatively, samples were decalcified for hematoxylin and eosin staining and p65 immunohistochemical staining.

### 4.4. Bone Marrow-Derived Osteoclast Induction

Bone marrow-derived cells were separated from the tibiae and femora of six-week-old male C57BL/6 mice, induced using α-MEM medium (Thermo Fisher Scientific, Waltham, MA, USA) containing 50 ng/mL M-CSF (PeproTech, Rocky Hill, NJ, USA) for 3 days to differentiate into osteoclast precursors, and then induced into mature osteoclasts by treatment with 30 ng/mL RANKL (R&D Systems, Minneapolis, MN, USA) and 50 ng/mL M-CSF for another 4 days.

### 4.5. Tartrate-Resistant Acid Phosphatase (TRAP) Staining

TRAP staining was performed using a Leukocyte Acid Phosphatase (TRAP) kit (Sigma-Aldrich, St. Louis, MO, USA) according to the manufacturer’s instructions. Briefly, slides were fixed by immersion in fixative solution for 30 s, TRAP staining solution was prepared, and the slides were incubated for 1 h at 37 °C in the dark. Cells were observed using a light microscope, and osteoclasts are defined as TRAP-positive cells containing more than three nuclei.

### 4.6. Hydroxyapatite-Coated Plate Resorption Assay

Osteoclast bone-resorption ability was measured using hydroxyapatite-coated OsteoAssay plate resorption assays. Bone marrow-derived cells were seeded in 24-well OsteoAssay plates and induced with 50 ng/mL M-CSF for 3 days to generate osteoclast precursors, then treated with 30 ng/mL RANKL and 50 ng/mL M-CSF for another 4 days. Cells were removing using sodium hypochlorite solution. The absorption pit aera were observed using a light microscope. Resorption pit aeras were quantified using ImageJ software (NIH, Bethesda, MD, USA).

### 4.7. Quantitative Real-Time Polymerase Chain Reaction (qPCR) 

Bone marrow-derived osteoclasts or RAW264.7 cells were lysed in TRIzol (Life Sciences, Grand Island, NY, USA) to extract total RNA. cDNA was synthesized using the Prime Script RT Master Mix kit (TaKaRa, Kusatsu, Shiga, Japan). The expression levels of osteoclastogenesis-related genes, *NFATc1, TRAP, OSCAR, MMP-9*, and *CTSK* were measured using qPCR with Power SYBR Green Master Mix (Life Sciences, Grand Island, NY, USA), and the mRNA expression of the above target genes were normalized to the mRNA levels of β-actin. The primer sequences used are listed in Table 1.

### 4.8. Subcellular Fractionation and Western Blotting

For subcellular fractionation, nuclear and cytoplasmic fractions were obtained using NE-PERTM Nuclear and Cytoplasmic Extraction Reagents (Thermo Fisher Scientific, Waltham, MA, USA) according to the manufacturer’s instructions. For traditional Western blotting, cells were lysed with radio-immunoprecipitation assay buffer. After protein quantification, the samples were separated using sodium dodecyl sulfate polyacrylamide gel electrophoresis on 10% gels and transferred to nitrocellulose membranes. The membranes were then blocked with 5% bovine serum albumin (BSA) in TBST and sequentially incubated with primary antibodies and horseradish peroxidase-linked secondary antibodies. The protein bands were visualized by enhanced chemiluminescence (Thermo Fisher Scientific, Waltham, MA, USA) and quantified by densitometry analysis using ImageJ software (NIH, Bethesda, MD, USA). The primary antibodies were used as follows: p-IκBα (Cell Signaling Technology, Beverly, CA, USA); IκBα (Cell Signaling Technology, Beverly, CA, USA); p-P65 (Cell Signaling Technology); P65 (Cell Signaling Technology, Beverly, CA, USA); β-actin (Cell Signaling Technology, Beverly, CA, USA); Lamin B (Proteintech, Shanghai, China); GAPDH (Cell Signaling Technology, Beverly, CA, USA).

### 4.9. Immunofluorescence 

Bone marrow-derived cells were cultured on coverslips, seeded in 24-well plates and induced with 50 ng/mL M-CSF to generate osteoclast precursors. The cells were then stimulated with 30 ng/mL RANKL for 30 min and fixed in paraformaldehyde for 15 min. Triton-X-100 was used to permeabilize the cell membrane. Sections were then blocked in 5% BSA for 1 h and incubated with anti-P65 antibodies (Cell Signaling Technology, Beverly, CA, USA) at 4 °C overnight in the dark. The cells were then incubated with secondary antibodies labeled with Alexa Fluor 488 (Thermo Fisher Scientific, Waltham, MA, USA) and 4′,6-diamidino-2-phenylindole (Thermo Fisher Scientific, Waltham, MA, USA). P65 location was observed using a confocal microscope. 

### 4.10. Luciferase Reporter Gene Assay 

RAW264.7 cell line was gifted by professor Qiming Liang (Shanghai Jiao Tong University School of Medicine). RAW264.7 cells were plated in 24-well plates and transfected with pGL4.32 (luc2P/NF-κB-RE/Hygro) plasmid (Promega, Madison, WI, USA) and R-luc plasmid (Promega, Madison, WI, USA) for 24 h, followed by incubation with Salubrinal (10 µM) or BAY-11 (10 µM) for 24 h, then stimulated with RANKL (100 ng/ml) for 30 min, Luciferase activity was detected using a dual-luciferase reporter assay system (Promega, Madison, WI, USA) and a multicell plate luminometer according to the manufacturer’s instructions. 

### 4.11. RAW264.7 Cell-Derived Osteoclast Induction and siRNA Transfection

Two pairs of siRNAs were designed against mouse P65 using following siRNA sequences: 

siRNA1 sense, 5′-GGACCUAUGAGACCUUCAATT-3′ and siRNA1 antisense, 5′-UUGAAGGUCUCAUAGGUCCTT-3′; siRNA2 sense, 5′-CCAUGGAGUUCCAGUACUUTT-3′ and siRNA2 antisense, 5′-AAGUACUGGAACUCCAUGGGC-3′. RAW264.7 cells were transfected with siRNA using Attractene Transfection Reagent (Qiagen, Germantown, MD, USA) for 24 h, then induced with 100 ng/mL RANKL for 24 h, subsequently plated in 96-well plates, and induced with 100 ng/mL RANKL for another 72 h.

### 4.12. Statistical Analysis 

The data are presented as means ± SEM and were analyzed using PRISM (version 6.0, GraphPad Software, Inc., San Diego, CA, USA). Statistical analysis was performed using a two-tailed Student’ *t*-test and *p*-values < 0.05 were considered to be statistically significant. 

## Figures and Tables

**Table 1:** Primer sequences for quantitative Real-time Polymerase Chain Reaction (qPCR).

Name	5′–3′	Sequence
*NFATc1*	Forward	CGGTAACACCACCCAGTATACC
	Reverse	GACTTGATAGGGACCCCATCAC
*TRAP*	Forward	CCAATGCCAAAGAGATCGCC
	Reverse	TCTGTGCAGAGACGTTGCCAAG
*OSCAR*	Forward	GGGGTGACAAGGCCACTTTT
	Reverse	CTGGACAGCCAGACACTAAAG
*MMP-9*	Forward	CTGGACAGCCAGACACTAAAG
	Reverse	CTCGCGGCAAGTCTTCAGAG
*CTSK*	Forward	GACGCAGCGATGCTAACTAA
	Reverse	CCAGCACAGAGTCCACAACT
*β-actin*	Forward	TGTCCACCTTCCAGCAGATGT
	Reverse	AGCTCAGTAACAGTCCGCCTAG

## Data Availability

The data presented in this study are available in the article.

## Abbreviations

RA	Rheumatoid arthritis
CIA	Collagen-induced arthritis
DMARDs	Disease-modifying antirheumatic drugs
M-CSF	Macrophage-colony stimulating factor
RANKL	Receptor activator of nuclear factor-κB ligand
MMP	Matrix metalloproteinase
NFATc1	Nuclear factor of activated T-cells, cytoplasmic, calcineurin dependent 1
TRAP	Tartrate-resistant acid phosphatase
CTSK	Cathepsin K
OSCAR	Osteoclast-associated receptor
CAIA	Anti-collagen antibody-induced arthritis
AP-1	Activating protein-1
CHX	Cycloheximide
PBS	Phosphate-buffered saline
BSA	Bovine serum albumin
